# Molecular profiles of BRCA1-mutated and matched sporadic breast tumours: relation with clinico-pathological features

**DOI:** 10.1054/bjoc.2001.1937

**Published:** 2001-08

**Authors:** E M J J Berns, I L van Staveren, L Verhoog, A M W van de Ouweland, M Meijer-van Gelder, H Meijers-Heijboer, H Portengen, J A Foekens, L C J Dorssers, J G M Klijn

**Affiliations:** Division of Endocrine Oncology, Department of Medical Oncology, Rotterdam Cancer Center (Daniel den Hoed Kliniek) and University Hospital Rotterdam; Department of Pathology and, Erasmus University Rotterdam, The Netherlands; Department of Clinical Genetics, Erasmus University Rotterdam, The Netherlands

**Keywords:** BRCA1, cDNA array, breast cancer, clinical features, molecular profiles

## Abstract

About 5–10% of breast cancers are hereditary; a genetically and clinically heterogeneous disease in which several susceptibility genes, including BRCA1, have been identified. While distinct tumour features can be used to estimate the likelihood that a breast tumour is caused by a BRCA1 germline mutation it is not yet possible to categorize a BRCA1 mutated tumour. The aim of the present study is to molecularly classify BRCA1 mutated breast cancers by resolving gene expression patterns of BRCA1 and matched sporadic surgical breast tumour specimens. The expression profiles of 6 frozen breast tumour tissues with a proven BRCA1 gene mutation were weighed against those from 12 patients without a known family history but who had similar clinico-pathological characteristics. In addition two fibroblast cultures, the breast cancer cell-line HCC1937 and its corresponding B-lymphoblastoid cell line (heterozygous for mutation BRCA1 5382insC) and an epithelial ovarian cancer cell line (A2780) were studied. Using a high density membrane based array for screening of RNA isolated from these samples and standard algorithms and software, we were able to distinguish subgroups of sporadic cases and a group consisting mainly of BRCA1-mutated breast tumours. Furthermore this pilot analysis revealed a gene cluster that differentially expressed genes related to cell substrate formation, adhesion, migration and cell organization in BRCA1-mutated tumours compared to sporadic breast tumours. © 2001 Cancer Research Campaign http://www.bjcancer.com


*British Journal of Cancer* (2001) **85**(4), 538–545
© 2001 Cancer Research Campaign
doi: 10.1054/ bjoc.2001.1937, available online at http://www.idealibrary.com on IDEAL

### Molecular profiles of BRCA1-mutated and matched sporadic breast tumours: relation with clinico-pathological features

**EMJJ Berns[1], IL van Staveren[1], L Verhoog[1,2], AMW van de Ouweland[3], M Meijer-van Gelder[1], H Meijers-Heijboer[3], H Portengen[1], JA Foekens[1], LCJ Dorssers[2] and JGM Klijn[1]**

[1]Division of Endocrine Oncology, Department of Medical Oncology, Rotterdam Cancer Center (Daniel den Hoed Kliniek) and University Hospital Rotterdam; [2]Department of Pathology and [3]Department of Clinical Genetics, Erasmus University Rotterdam, The Netherlands

**Summary** About 5–10% of breast cancers are hereditary; a genetically and clinically heterogeneous disease in which several susceptibility genes, including BRCA1, have been identified. While distinct tumour features can be used to estimate the likelihood that a breast tumour is caused by a BRCA1 germline mutation it is not yet possible to categorize a BRCA1 mutated tumour. The aim of the present study is to molecularly classify BRCA1 mutated breast cancers by resolving gene expression patterns of BRCA1 and matched sporadic surgical breast tumour specimens. The expression profiles of 6 frozen breast tumour tissues with a proven BRCA1 gene mutation were weighed against those from 12 patients without a known family history but who had similar clinico-pathological characteristics. In addition two fibroblast cultures, the breast cancer cell-line HCC1937 and its corresponding B-lymphoblastoid cell line (heterozygous for mutation BRCA1 5382insC) and an epithelial ovarian cancer cell line (A2780) were studied. Using a high density membrane based array for screening of RNA isolated from these samples and standard algorithms and software, we were able to distinguish subgroups of sporadic cases and a group consisting mainly of BRCA1-mutated breast tumours. Furthermore this pilot analysis revealed a gene cluster that differentially expressed genes related to cell substrate formation, adhesion, migration and cell organization in BRCA1-mutated tumours compared to sporadic breast tumours.
© 2001 Cancer Research Campaign http://www.bjcancer.com

**Keywords**: BRCA1; cDNA array; breast cancer; clinical features; molecular profiles

Of all cancer types breast cancer is the most common malignancy in females. Women in Western countries have a lifetime risk of about 10% to develop breast cancer and it is the leading cause of death among women at 35–55 years of age (Harris et al, 1992). About 5–10% of breast cancers are hereditary; a genetically and clinically heterogeneous disease in which several susceptibility genes, including BRCA1 and BRCA2, have been identified (reviewed by Martin and Weber, 2000; Alberg and Helzlsouer, 1997; Paterson, 1998).

BRCA1 encodes for a multifunctional protein which contributes to homologous recombination and DNA damage repair, cell cycle checkpoint control, embryonic proliferation, transcription regulation and ubiquitination (reviewed by Deng and Scott, 2000; Welcsh et al, 2000). The majority of the BRCA1 mutations result in premature chain termination during protein translation, and these are scattered over the entire coding region of the BRCA1 gene. Hence, inactivation of BRCA1 has been proposed to induce a mutator phenotype which would accelerate tumour growth by increasing genetic instability. Compared to sporadic cases, inherited breast cancer cases from germline mutations in BRCA1 have a number of distinctive clinical features such as an early age of onset, a higher prevalence of bilateral breast cancer and the presence of associated tumours, in particular ovarian cancer. Breast tumours from BRCA1-carriers are more likely to be highly proliferative and poorly differentiated (grade 3), also more frequently have an atypical medullary-like appearance (Lakhani et al, 1998) with a higher degree of lymphocyte infiltration and show an excess of continuous pushing margins. Moreover these tumours are more often oestrogen (ER) and progesterone (PgR) receptor and cERBB2 negative and frequently show TP53 alterations, reviewed by (Phillips et al, 1999). While these tumour features can be used to estimate the likelihood that a breast tumour is caused by a BRCA1 germline mutation it is not yet possible to distinguish a BRCA1 mutated tumour by basic pathology.

Gene expression analysis by cDNA (micro)arrays is a powerful tool for characterizing the variation in transcriptional programs in cells and tissues. Expression profiles of genes using mRNA from breast tumour cell lines, grossly dissected tumours (Perou et al, 1999) or from laser capture microdissected cells from human breast tumour tissues (Sgroi et al, 1999) have been generated. The data revealed several clusters of co-expressed genes, some of which could be connected to other cell types including stromal cells and B lymphocytes. Although it is feasible to analyse gene expression in microdissected tumour specimens, Ross (Ross et al, 2000) and Perou (Perou et al, 2000) showed that it is possible to explore and to interpret some of the biology of surgical tumour tissues by analysing these tumours intact. Thus, as in conventional morphological pathology, one might be able to observe interactions between a tumour and its microenvironment in this way.

*Received 5 October 2000*
*Revised 14 March 2001*
*Accepted 27 April 2001*

*Correspondence to:* EMJJ Berns Department of Medical Oncology, Josephine Nefkens Institute, Room Be 424, P.O. Box 1738, 3000 DR Rotterdam, The Netherlands

Recent data imply that gene expression analysis with (micro)arrays allows for tumour classification (Golub et al, 1999) and possibly for prediction of prognosis (Alizadeh et al, 2000), while Bittner et al (2000) discovered a subset of genes critical for the metastatic process of melanomas. In accordance with these observations, a relevant (sub)set of genes expressed in hereditary cancers could aid in the distinction between BRCA1 gene mutated and sporadic breast tumours. The aim of the present study is to molecularly classify BRCA1 mutated breast cancers by resolving gene expression patterns of BRCA1 and matched sporadic surgical breast tumour specimens.

### MATERIALS AND METHODS

### Patients and samples

Tumour specimens were selected from a pool of frozen specimens (liquid nitrogen) originally submitted to our laboratory for steroid hormone receptor analyses. Seven tumours had a previously established germ-line mutation in the BRCA1 gene (F1-7). Of these seven BRCA1 mutations three occurred at the BRCT domain (2 times 5382insC and 5396 +1G>A in samples F3,5,7). This domain interacts with TP53 and functions as a transactivator in both TP53 dependent and independent ways. Sample F4 contained the large genomic deletion encompassing exon 13 that occurs quite frequently in the Dutch population. Two tumours had a 1411insT alteration in exon 11 of the BRCA1 gene (F2,6). The latter exon encodes for a domain that interacts with RAD 51 that is involved in DNA damage repair. The 185insA mutation, in the N-terminus of the BRCA1 gene, is within the RING finger domain. These BRCA1 mutated breast tumours were matched to 15 sporadic breast tumours from patients without a family history of breast cancer (S1–15), for steroid hormone receptor status, tumour size, differentiation grade, histology, age, menopausal and nodal status of the patient. The information on family history was based on retrospective chart review (the charts are computerized and updated on a regular basis). The pathology data were extracted from pathology reports and, in addition, all slides were reviewed by a single pathologist. Other inclusion criteria were that frozen tumour sample weight needed to be > 300 mg, with epithelial tumour cells comprising at least 50% and available data on follow-up. The median age of the patients was 37 years (range, 26–73 years) at time of primary surgery. Sixteen of the 22 patients had breast-conserving therapy (lumpectomy) and six a modified mastectomy. Eight patients were node positive. Six patients (27%) received systemic adjuvant chemotherapy (cyclophosphamide, methotrexate and 5-fluorouracil). Three patients were post-menopausal. Tumour progression occurred in 12 patients and 8 patients died during follow-up. Of these 22 tumours, 19 had a poor differentiation grade, 15 were larger than 2 cm (T2/T3), 15 were ER negative and 11 had a TP53 mutation. Our study design was approved by the medical ethical committee of the University Hospital Rotterdam, the Netherlands and by our Cancer Centre (protocol DDHK 91–17, updated in 1995).

To establish a context for the interpretation of the variations in expression patterns seen in the tumour samples we also included two fibroblast cultures, the epithelial breast cancer cell line HCC1937 (hemizygous for the BRCA1 5382insC mutation) and its matching B-lymphoblastoid cell line (heterozygous for this mutation; Tomlinson et al, 1998), and the epithelial ovarian cancer cell line A2780.

### Assays of ER and PgR and analyses of TP53 and BRCA1 gene status

ER and PgR levels were determined in cytoplasmic extracts (cytosols), routinely prepared according to procedures recommended by the EORTC Breast Cancer Cooperative Group, with ligand binding assays or with enzyme immunoassays (ER-EIA and PgR-EIA, Abbott Laboratories, IL, USA), as described previously (Foekens et al, 1989). For the study of TP53 mutations, exons 5–8 of the TP53 gene were analysed with SSCP and direct sequencing and with immunological techniques, i.e. immunohistochemical analyses using MAB 1801 and DO1 and the luminometric immuno assay (LIA), as described previously (Berns et al, 1996; Kuenen-Boumeester et al, 1999; Berns et al, 1998). Samples with mutations observed by sequence analysis are designated as ‘mutated’ whereas samples in which an over-expression in the tumour cells for both the anti-p53 mABs 1801 and DO1 was observed, but without a mutation in exons 5–8, are designated as ‘IH > 80%’, i.e. altered (listed in Table 1). Twelve out of 23 samples are wild-type, a mutation was observed in 8 samples and an over-expression (>80% of the tumour cells stained positive) without mutation in exon 5–8 was observed in three cases. The majority of the frozen sporadic breast tumours were tested for BRCA1 protein expression, and BRCA1 gene alterations by DSDI with subsequent sequencing of the altered BRCA1 gene fragment (Papelard et al, 2000) and all samples were tested for 22 different mutations, by allele specific oligonucleotide hybridization for distinct mutations or by PCR for exon deletions, with a minor modification, as described previously (Petrij-Bosch et al, 1997; Peelen et al, 1997). This targeted analysis included the Dutch founder mutations in BRCA1 and BRCA2 that at present account for 60−70% of the germ-line mutations, i.e. 185insA, 185delAG, 1411insT, 1438delT, 2312del5, 2329delC, Q780X, 2765delTGC, 2804delAA, E908X, 2846del4, 2883del4, E1214X, 3938insG, Q1281X, IVS12-1643del3835 (3.8 kb deletion of exon 13), 5396 + 1 G > A, 5382insC, IVS21-36del510, IVS22 + 5G > A (deletion of exon 22) in BRCA1, 5579insA and 6503delTT in BRCA2. With respect to the sporadic tumours on 10 of the 15 samples a full sequencing was done of the BRCA1 gene. All samples were found to be wild-type, although in tumour sample S8 a polymorphism (S1436S, see Table 1) was observed. Targeted BRCA1 mutation analyses in the remaining five samples revealed no alterations.

### RNA isolation, cDNA generation and array hybridization

RNA was prepared from the frozen surgical tumour samples under stringent conditions to avoid degradation and contamination. Briefly: 26 frozen tissue sections, 50 μm each, were cut with a cryostat at – 20˚C. The first and last sections (5 μm) were counter-stained with H&E and the percentage of epithelial tumour cells was estimated. Total RNA from tumour samples and cell lines was extracted using RNAZolB (Campro Scientific, Veenendaal, The Netherlands) described by Auffray (Auffray and Rougeon, 1980) with a median total RNA recovery of 203 μg (range 87–427 μg). RNA integrity was verified by agarose gel electrophoresis. The RNA samples were treated with DNAse and genomic PCR analyses, with intronic primers, for TP53 confirmed that no residual DNA was present. These RNA samples (2–5 μg) were used to synthesize $^{32}$P-radiolabelled complex cDNA probes and these fragments were hybridized overnight to the Clontech Atlas

© 2001 Cancer Research Campaign

**Table 1** Patient and tumour characteristics arranged according to the outcome of the clustering analysis

| Tumour sample | Group A | | | | | | | Group B | | | | | | | | | | | |
|---|---|---|---|---|---|---|---|---|---|---|---|---|---|---|---|---|---|---|---|
| | **S10** | **F1** | **S1** | **S9** | **S8** | **S11** | **S4** | **S13** | **S12** | **F5** | **F3** | **F4** | **F2** | **S3** | **S2** | **F6** | **S15** | **S14** | **HCC** |
| BRCA-mutation | wt | 185insA | – | wt | wt(p) | – | – | wt | wt | 5382insC | 5396+1G> | del3.8 kb | 1411insT | wt | wt | 1411insT | wt | wt | 5382insC |
| AGE at diagnosis | 30–40 | 40–50 | 20–30 | 20–30 | 30–40 | 30–40 | 30–40 | 30–40 | 30–40 | 40–50 | 30–40 | 50–60 | 40–50 | 30–40 | 30–40 | 30–40 | 30–40 | 30–40 | 20–30 |
| Tumour size | T2 | T1 | T2 | T2 | T1 | T2 | T2 | T1 | T2 | T1 | T1 | u | T2 | T2 | T3 | T2 | T2 | T2 | u |
| Differentiation grade | poor | moderate | moderate | poor | poor | poor | poor | poor | poor | poor | poor | poor | poor | poor | poor | poor | poor | poor | poor |
| Nodal status | N0 | N0 | N0 | N1 | N0 | N0 | N0 | N0 | N0 | N0 | N0 | N1 | N0 | N0 | N1 | N1 | N1 | N1 | N0 |
| Histology | med | idc | idlc/dcis | idc | med | idc | idc | idc | idc | idc | idc | idc | idc/dcis | idc | idc | idc | medul | idc | idc |
| Surgery | lumpect | ablatio | lumpect | lumpect | lumpect | lumpect | lumpect | lumpect | lumpect | lumpect | lumpect | ablatio | ablatio | lumpect | ablatio | lumpect | lumpect | lumpect | ablatio |
| Adjuvant chemo. | no | n-anthra | no | n-anthra | no | no | no | no | no | no | no | n-anthra | no | no | n-anthra | n-anthra | no | no | u |
| Metastasis | no | 2nd prim | Bone | meta | no | no | lung LRR | bone LLR | LRR | LLR+ | no | u | visc | no | no | visc | no | meta | u |
| MFS-months | 46 | u | 38 | 14 | 110 | 109 | 25 | 21 | 108 | 39 | 66 | u | u | 72 | 132 | 22 | 76 | 14 | u |
| Alive/death-months | A > 46 | A > 58 | D-50 | D-23 | A > 110 | A > 109 | D-36 | D-31 | A > 108 | A > 83 | A > 66 | A > 76 | A > 39 | A > 72 | A > 132 | D-30 | A>76 | D-15 | u |
| Double primary | no | yes | mam2 | no | no | no | no | no | mam2 | no | no | no | no | no | no | yes | no | no | u |
| ER | 15 | – | 82 | 18 | 0 | 0 | 0 | 4 | 3 | 47 | 1 | 10 | 0 | 3 | 0 | u | 8 | 0 | L |
| PR | 0 | – | 401 | 173 | 9 | 0 | 6 | 7 | 7 | 0 | 1 | 3 | 0 | 8 | 10 | u | 10 | 21 | L |
| TP53 status | mutated | wt | wt | mutated | wt | wt | wt | mutated | wt | mutated | wt | wt | IH > 80% | wt | IH>80% | wt | wt | mutated | mutated |
| % tumour cells | 60 | 75 | 60 70 | 80 70 | 90 95 | 80 | 70 25 | 90 80 | 80– 90 | > 75 | > 80 | 70 | 60 50 | 50 40 | 90 60 | 80 | 60 | 80 | 100 |

BRCA1, wt: wild type; T1, tumour size below 2 cm; T2, between 2 and 5 cm; T3, larger than 5 cm. NO, node negative; N1, node positive. Histology, idc: invasive ductal carcinoma, med: medullary carcinoma, dcis: ductal carcinoma in situ. ER and PgR values below 10 fmol/mg protein were considered negative (N), between 10 and 75 fmol/mg protein as intermediate (I), and above 75 fmol/mg protein as high (H). TP53 status is considered 'mutated' or 'over-expressed' based on criteria described in the methods section. Percentage(s) of tumour cells at start and finish of sectioning of the samples are listed.

*© 2001 Cancer Research Campaign*

Human Cancer cDNA expression arrays according to the manufacturer's instructions (Clontech Corp., Palo Alto, CA; http://www.clontech.com). These filters contain 588 cancer related genes organized by their functional classes that are spotted in duplicate. Membranes were subsequently washed and exposed to a Phosphorlmaging screen for 1–7 days.

### Array data analysis

Data were acquired and quantified using the Molecular Dynamics Phospholmager and ImageQuant software (Molecular Dynamics, Sunnyvale, CA, USA). The signals from the duplicate spots and their reproducibility was verified by comparing these duplicate spots using the AtlasImage V1.0 software (Clontech Corp., Palo Alto, CA). The mean value and percentual deviation of the duplicate spots was calculated. Subsequently, the median deviation of 20 randomly duplicate spots on each filter was estimated. The data on the duplicate spots were highly reproducible since the overall median value of the percentual deviation between these duplicate spots of all filters was only 2% (range: 0.4–9%). When applicable the data of the individual spots were corrected for excess hybridization ('bleeding') of adjacent overpressed genes. The background of each filter was determined by measuring the level (amount of counts) in several 'blank' areas (i.e., no genes were spotted in those areas) of the filter. Signals that were on average 1.5 times background level were considered too low for an accurate measurement and were excluded from further analysis, thus defined as negative/missing. As a consequence only those genes with a 1.5 times expression above the background were considered positive hybridizations and these were included in further array data analyses. Moreover, when on the filter the expression of half or more of the genes was below 1.5 times the background level (i.e. negative/too low), the hybridization was considered as poor and hence four arrays were excluded from further analyses. In summary the criteria for inclusion of expression data in the final analyses were that: (a) individual signal of a gene had to be greater than 1.5 times that of the background level (defined as positive hybridizations); (b) each tumour sample must show proper hybridization, meaning that it had to be above 1.5 times the background, for at least 50% of all 588 genes analysed; and (c) each single gene needed to be expressed above 1.5 times the background in at least 14 of the 18 breast tumour samples studied. HER2/neu was for example excluded since it was expressed above 1.5 times the background in only 8 out of 18 samples and FGF3/INT2 showed a positive hybridization in only 4 out of 18 samples.

Hierarchical clustering was applied to the samples and the genes using the cluster analysis software developed by Eisen (Eisen et al, 1998) (*http://rana.stanford.edu/clustering*) on spots that were corrected for background. According to the recommendations, hierarchical clustering was performed on the log-transformed and normalized data set using an average linkage clustering and Spearman rank correlation as similarity metric. Data are available upon request. The resulting outcome was displayed with the TreeView program (Eisen et al, 1998). The log-transformed data of each gene relative to its median expression level were visualized as a colour: red represented expression above the median, black represented the median and green indicated expression less than the median. The colour intensities indicate the magnitude of the deviation from the median and reflect the level of expression. Grey specified gene expression data that were missing (see above). Rows represented genes and columns the experimental samples.

### RESULTS

### Tumours and arrays

The expression profiles (portraits) of frozen breast tumour tissues with a proven BRCA1 gene mutation (F) were weighed against those from patients without a known family history (S) and a BRCA1 mutation but which had similar clinico-pathological characteristics. For this exploratory analysis we used the Atlas Human Cancer cDNA Expression Arrays. Four samples, out of the initial 22 breast tumour samples studied, were excluded since the hybridization results did not fulfil the criteria, i.e. not above 1.5 times the background for at least 50% of all 588 genes analysed (samples F7, S5–7). Thus the profiles of 6 frozen breast tumour tissues with a proven BRCA1 gene mutation and of 12 samples without BRCA1 gene mutation and of 1 control cell-line, HCC1937, were weighed. In order to identify patterns of gene expression in the remaining 18 breast tumour specimens (listed in Table 1) we have used the hierarchical clustering method. Of the 588 genes studied, 248 were not included in the final analysis since these genes did not show a hybridization level of 1.5 times above the background in 14 of the 18 tumour samples analysed (Eisen et al, 1998). The overall similarity in gene expression patterns of this basic set generates a dendrogram of tumour specimens in which the pattern and length of the branches reflects the relatedness of the samples. Two differentially expressed genes, integrin beta 4 and HPRT were validated using a different methodology, i.e. RT-PCR on a subset of tumours, and these RT-PCR results confirmed the outcome of the cDNA array data (not shown).

### Clustering of tumours

Each tumour showed a remarkable variation in its gene expression pattern, as seen in Figure 1. Two distinct main groups with close overall similarity were detectable. The first group, including seven samples, i.e. S1, 4,8–11 and F1, consisted primarily of sporadic tumours (Figure 1, tumour group A). Sample 'F1' was a second tumour from a BRCA1 mutation carrier in the contralateral breast (see Table 1). This patient received adjuvant chemotherapy for the primary tumour which may have altered the characteristics of the analysed second primary tumour. The second closely related group of tumours based on the lengths of the branches of the dendrogram, i.e. F2-6 and S2 + 3, consisted mainly of BRCA1 mutated tumours. Samples S12 + 13 and S14 + 15 were, although less related, both attached to this less homogeneous cluster (Figure 1 and Table 1, tumour group B). Group B also contained the epithelial breast tumour cell-line HCC1937, which is hemizygous for the BRCA1 mutation 5382insC.

Subsequently, patient and tumour characteristics were related to the outcome of the clustering. The data are listed in Table 1 and are given according to the outcome of the dendrogram shown in Figure 1. Group A represents tumours from patients that were mainly node negative (NO, 6 out of 7 patients) whereas 5 out of 11 patients were node positive (N +) in group B. There was no difference between the medium time of follow-up of patients alive in both subgroups (83 months in group A versus 76 months in group B) or the occurrence of metastasis or double primaries. Less patients were alive in group A (4 out of 7 patients) compared to group B (8 out of 11 patients, Table 1). With respect to tumour characteristics, the TP53 gene was more often altered in group B

© 2001 Cancer Research Campaign

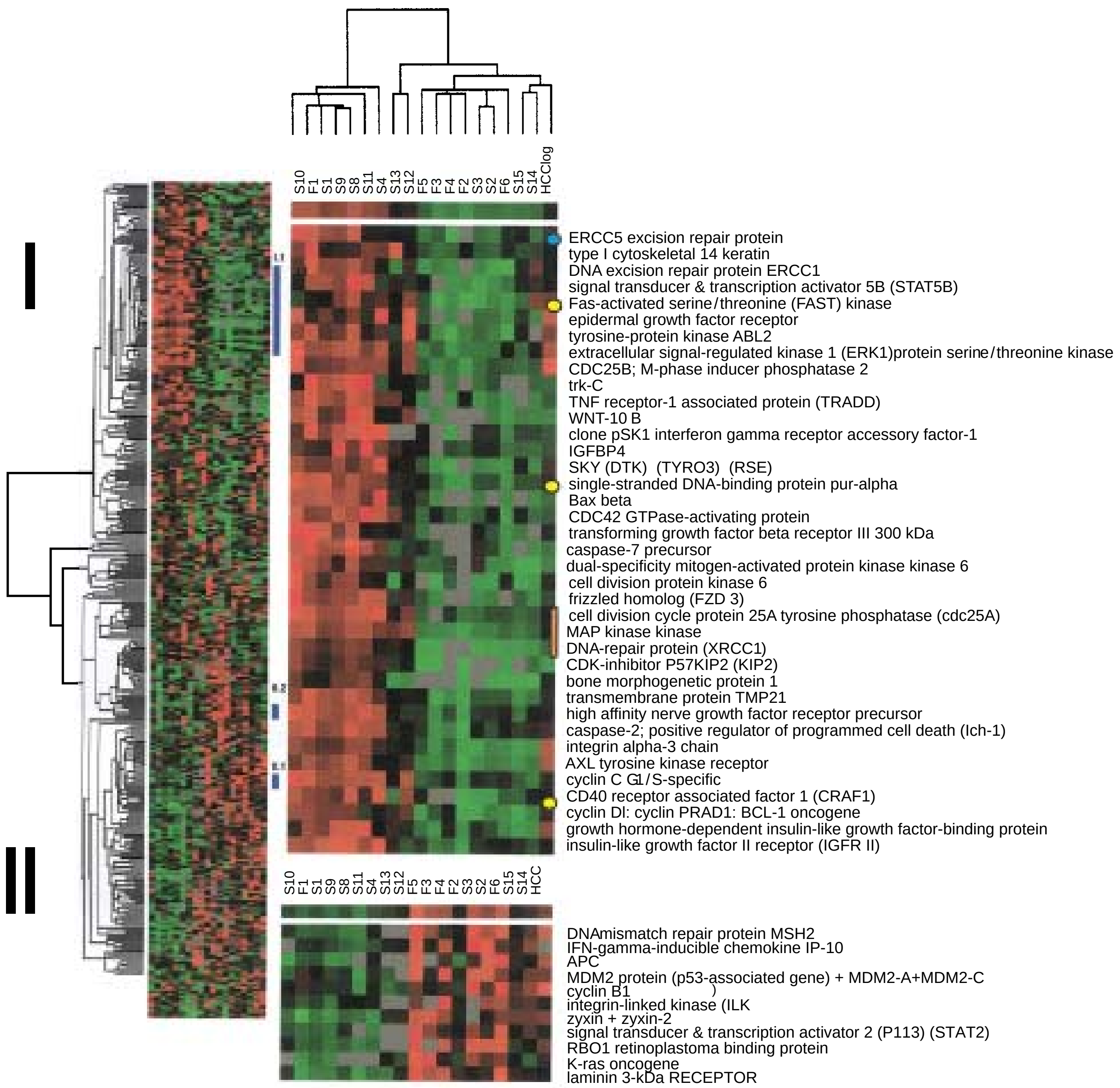

**Figure 1** Overview of gene expression profiles of BRCA1 mutated and sporadic breast tumours. Left panel shows the relative expression levels of 340 genes, given in rows, from 19 samples, in columns, in a cluster diagram overview. (F1–6; six tumour samples with a BRCA1 mutation, HCC(log): an epithelial tumor cell-line, HCC1937 grown at confluence, with a BRCA1 mutation and S1–5 and 8–15: 12 sporadic tumour samples). The expression of log transformed data of each gene relative to its median expression level is visualized as a colour: red representing expression greater than the median, green representing expression less than the median, black representing the median. Grey indicates technically inadequate or background data. The colour intensities represent the magnitude of the deviation from the median and reflect the level of expression. The distinctive molecular profiles of each tumour provides a basis for sub-classification. The hierarchical clustering, which depicts genes and samples according to similarity in their overall expression patterns, reveals the tissue samples in sub-groups of sporadic cases and a group that mainly consists of BRCA1 mutated cases, i.e. tumours F2–6. An expanded view of two sets of differentially expressed genes, i.e. cluster I.1 and cluster II.1 is shown in panels at the right. The marked genes (CK14, EGF-R, Bax, Cyclin D1, and XRCC1, p57, MAPKK) are discussed in more detail

(5 out of 11 tumours in group B compared with 2 out of 7 tumours in group A). None of the other clinico-pathological factors were related with the clustering of the tumours (Table 1).

### Subsets of clustered genes

The genes were also divided into two main clusters. Cluster I depicts genes that were relatively highly expressed in tumour group A and expressed at a lower level in group B (see Figure 1). Within group I a small subset of genes was chosen which showed comparable expression profiles in all samples (correlation coefficient = 0.77). This small cluster, designated 1.1, contains 36 of the 340 displayed genes. It comprises many genes whose products are related to or necessary for DNA damage response and repair (i.e. XRCC1, ERCC1&5, ssDNA binding protein our alpha), for apoptosis (Bax, caspases 2&7, TRADD, CRAF1, FAST kinase) or for cell cycle and growth regulation (MAPK 1, MAPK/ERK kinases 1 and -6, MEKK, p57KIP2, STAT5B, cdc25A and -B, cyclins D1 and C).

 *© 2001 Cancer Research Campaign*

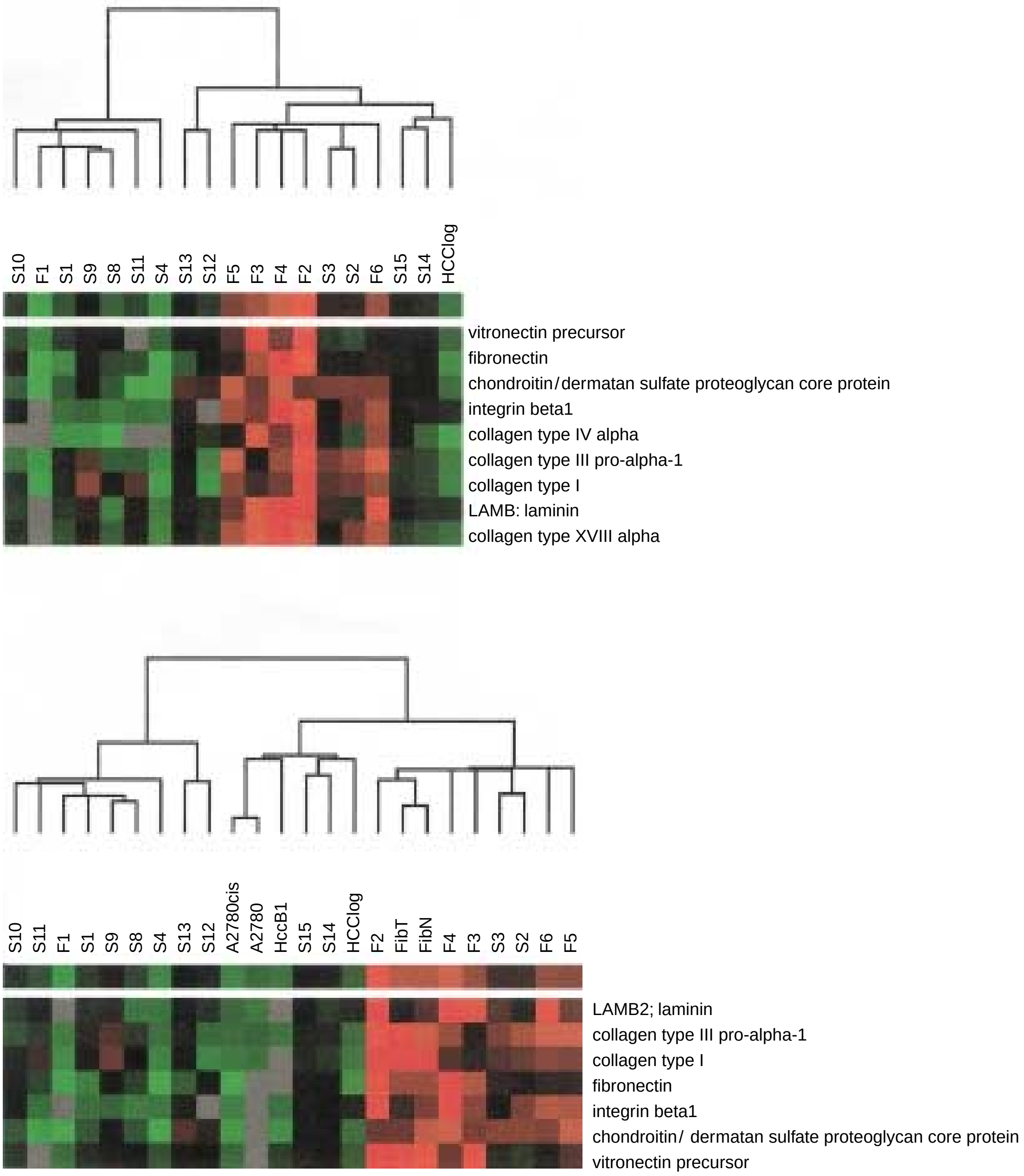

**Figure 2** An expanded view of a set of differentially expressed genes in the BRCA1 mutated tumours is shown for the samples included in Figure 1 and for all samples tested. Upper panel shows an expanded view of cluster II.2, containing genes with relatively high expression levels in BRCA1 mutated tumours, from Figure 1. To provide a tissue type framework for the analysis of the variations in expression patterns, two fibroblast cultures, the epithelial breast cancer cell line HCC1937 and its corresponding B-lymphoblastoid cell line and an epithelial ovarian cancer cell line (A2780, which has also been cultured in the presence of cisplatin; A2780cis) were included in the advanced analysis. The overall characteristics of this clustering were very similar to those of Figure 1 and only minor changes in genes and tumour samples were observed. The corresponding gene cluster section is shown in the lower panel

Furthermore, receptors (IGFIIR, EGFR, IGFBP4, TGFbetaRIII) and several cell fate and development related genes were relatively over-expressed in this cluster. Within this cluster 1.1 the DNA repair gene XRCC1, the cyclin dependent inhibitor p57KIP2 and the MAP kinase kinase were closely correlated ($P$ = 0.95). The relatively high expression of the cytoskeletal 14 keratin in the tumour samples in group A points to a myoepithelial (basal) cell origin.

Cluster II comprises genes that were relatively lower expressed in tumour group A compared to group B. For example, the DNA mismatch repair gene MSH2, the tumour suppressor gene APC, the TP53 regulated protein MDM2, K-RAS and several invasion/adhesion associated genes were under-represented as compared to the B group (cluster II.1). Furthermore, we recognized a subset of genes that might characterise the BRCA1 mutated tumour types (Figure 2). This subset (correlation coefficient = 0.81; cluster 11.2) contains genes as vitronectin precursor, different collagens, laminin and fibronectin, which have been related to cell substrate formation, adhesion, migration and cell organization. To establish a context for the interpretation of the variations in expression patterns seen in the tumour samples, we additionally characterized two fibroblast cultures, the HCC1937

*© 2001 Cancer Research Campaign* 

corresponding B-lymphoblastoid cell line (heterozygous for mutation BRCA1 5382insC) and an epithelial ovarian cancer cell line (A2780). The outcome of this separate clustering analysis showed a slightly altered clustering of tumour samples and genes (Figure 2) but despite this suggested that the expression of the set of genes characteristic of adhesion and migration was a feature shared by these BRCA1 mutated tumour samples and the cultured fibroblasts. The percentage of epithelial tumour cells in the tumours in cluster A and B is, however, similar (Table 1). These data demonstrated that these BRCA1-tumour associated genes were higher expressed in fibroblast cultures but not in the epithelial ovarian and breast tumour cell lines (Figure 2).

### DISCUSSION

Breast cancer is a multifactorial and heterogeneous disease and further understanding of its biology is important. Many genes and signalling pathways controlling cell proliferation, differentiation and death, as well as genomic integrity have been reported to be involved. The aim of the present study was to use arrays as a tool to understand and classify BRCA1 mutated tumours based upon their global gene expression patterns. The expression profiles of six surgical frozen breast tumour samples and one epithelial cell-line from patients with an inherited BRCA1 gene mutation were compared with the profiles from 12 patients without a family history and without BRCA1 gene alterations. A hierarchical clustering method was used to group the variation in mRNA levels of genes.

We identified two major tumour groups. Group A represented tumours from patients that were mainly node negative and had fewer TP53 gene alterations with less patients alive during follow-up. Genes that were highly expressed in group A include cytokeratin 14, in which expression has been related to myoepithelial (basal) cells (Otterbach et al, 2000). Interestingly, this cluster also included genes whose expression patterns have been characterized by previous studies to be related to breast cancer prognosis (i.e. BAX, cyclin D1 and EGFR). Thus the EGF-receptor is highly expressed in this group of mainly ER-negative or ER-low cells. An inverse correlation of the level of EGFR and ER has been reviewed (Klijn et al, 1992). Recent data showed that in ER-negative or low cells NF-kappaB is increased by EGFR. The NF-kappaB on its turn transactivates the cell cycle regulator protein cyclin D1 which causes increased phosphorylation of RB, allowing for cell-cycle progression, more strongly in ER-negative cells (Biswas et al, 2000). This may indicate a specific group of ER-negative tumour cells with increased EGFR and cyclin D levels.

Interestingly, group B isolates five out of the six BRCA1 mutated tumours into a distinct sub-set. Only tumour F1 was positioned in group A. The latter tumour, however, was a second primary tumour from a BRCA1 mutation carrier (185insA) that emerged 57 months after primary surgery in the contralateral breast. The patient had recieved adjuvant chemotherapy (CMF) after surgery of the first primary tumour, which may have affected the characteristics of this second primary tumour. Tumours S2 and S3 that were also included in this sub-group did not show a BRCA1 mutation after complete sequence analysis. Breast cancers without a BRCA1 germline mutation but which nevertheless exhibit BRCA1 under-expression may have similar pathologic characteristics to breast cancers from BRCA1 germline mutation carriers (Wilson et al., 1999). We have therefore analysed the BRCA1 protein expression, using immunohistochemical techniques, but did not find any relation to the BRCA1 expression levels and clustering of the tumours. The BRCA2 gene hybridization data were extracted from the filter arrays and again no relation was observed between the expression levels of BRCA2 and clustering outcome. It is however still possible that gross genomic alterations, which cannot be detected by sequence analysis, have altered the BRCA1 gene or that BRCA2 gene mutations or mutations in other as yet undiscovered major breast cancer predisposing genes could be involved in these cases.

Examination of the gene expression profiles revealed a sub-set of genes may characterize the BRCA1 tumour types, i.e. vitronectin precursor, different collagens, laminins and fibronectin, which have been related to cell substrate formation, adhesion, migration and cell organization. Of these, fibronectin expression has been reported to be related to normal but not to mammary epithelial cells (Lee et al, 1991). Supplementary cluster analysis showed that these genes appear to be highly expressed in fibroblast but not in the two epithelial breast and ovarian tumour cell-lines. Thus expression of a set of genes characteristic of adhesion and migration was a feature shared by these BRCA1 mutated tumour samples and cultured fibroblasts. Interestingly, the expression of a set of genes characteristic of stromal cells, including collagen, has been reported to be a feature shared by breast tumour samples and the so as such described ‘stromal-like’ mammary tumour cell-lines Hs578T and BT549 (Perou et al, 2000). As a consequence, the authors suggested that the expression pattern seen in the tumour samples is likely to be due to the stromal component of the tumour. An explanation for the unexpected expression pattern observed in the present study could be the different growth patterns observed in BRCA1 associated tumours when compared to sporadic tumours, which is characterized by an excess of continuous pushing margins in the BRCA1 mutated tumours with fibroblasts that are highly active (reviewed by Philips et al, 1999). The latter finding will be a subject of future investigation. These markers may aid in the recognition of BRCA1 mutated tumours.

Although the sample size in any of the subgroups is too small to support firm statistical analyses, our preliminary data show the potential of gene expression based subtyping of breast tumours. Advanced gene expression profiling, recently used by Hedenfalk et al (2001), on glass cDNA arrays containing thousands of genes which allows for the internal calibration of the expression data, is preferred to identify the genes and profiles that matter for the molecular pathogenesis pathways of hereditary cancers. This may provide information on tumour response and possibly new targets for breast cancer prevention and therapy.

### ACKNOWLEDGEMENTS

The authors wish to thank M. Hoogenboom and Dr P Devilee at the LUMC, Leiden, The Netherlands for their valuable contribution to the sequence analyses of the BRCA1 gene.

### REFERENCES

Alberg AJ and Helzlsouer KJ (1997) Epidemiology, prevention, and early detection of breast cancer. *Curr Opin Oncol* **9**: 505–511

Alizadeh AA, Eisen MB, Davis RE, Ma C, Lossos IS, Rosenwald A, Boldrick JC, Sabet H, Tran T, Yu X, Powell JI, Yang L, Marti GE, Moore T, Hudson J Jr, Lu L, Lewis DB, Tibshirani R, Sherlock G, Chan WC, Greiner TC, Weisenburger DD, Armitage JO, Warnke R and Staudt LM (2000) Distinct types of diffuse large B-cell lymphoma identified by gene expression profiling. *Nature* **403**: 503–511

Auffray C and Rougeon F (1980) Purification of mouse immunoglobulin heavy-

© 2001 Cancer Research Campaign chain messenger RNAs from total myeloma tumor RNA. *Eur J Biochem* **107**: 303–314

Berns EM, Klijn JG, Smid M, van Staveren IL, Look MP, van Putten WL and Foekens JA (1996) TP53 and MYC gene alterations independently predict poor prognosis in breast cancer patients. *Genes Chromosomes Cancer* **16**: 170–179

Berns EM, van Staveren IL, Look MP, Smid M, Klijn JG and Foekens JA (1998) Mutations in residues of TP53 that directly contact DNA predict poor outcome in human primary breast cancer. *Br J Cancer* **77**: 1130–1136

Biswas DK, Cruz AP, Gansberger E and Pardee AB (2000) Epidermal growth factor-induced nuclear factor kappa B activation: A major pathway of cell-cycle progression in estrogen-receptor negative breast cancer cells. *Proc Natl Acad Sci USA* **97**: 8542–8547

Bittner M, Meltzer P, Chen Y, Jiang Y, Seftor E, Hendrix M, Radmacher M, Simon R, Yakhini Z, Ben-Dor A, Sampas N, Dougherty E, Wang E, Marincola F, Gooden C, Lueders J, Glatfelter A, Pollock P, Carpten J, Gillanders E, Leja D, Dietrich K, Beaudry C, Berens M, Alberts D and Sondak V (2000) Molecular classification of cutaneous malignant melanoma by gene expression profiling. *Nature* **406**: 536–540

Deng CX and Scott F (2000) Role of the tumor suppressor gene Brca 1 in genetic stability and mammary gland tumor formation. *Oncogene* **19**: 1059–1064

Eisen MB, Spellman PT, Brown PO and Botstein D (1998) Cluster analysis and display of genome-wide expression patterns. *Proc Natl Acad Sci USA* **95**: 14863–14868

Foekens JA, Portengen H, van Putten WL, Peters HA, Krijnen HL, Alexieva-Figusch J and Klijn JG (1989) Prognostic value of estrogen and progesterone receptors measured by enzyme immunoassays in human breast tumor cytosols. *Cancer Res* **49**: 5823–5828

Golub TR, Slonim DK, Tamayo P, Huard C, Gaasenbeek M, Mesirov JP, Coller H, Loh ML, Downing JR, Caligiuri MA, Bloomfield CD and Lander ES (1999) Molecular classification of cancer: class discovery and class prediction by gene expression monitoring. *Science* **286**: 531–537

Harris AL, Nicholson S, Sainbury R, Wright C and Farndon J (1992) Epidermal growth factor receptor and other oncogenes as prognostic markers. *J Natl Cancer Inst Monogr* **11**: 181–187

Klijn JG, Berns PM, Schmitz PI and Foekens JA (1992) The clinical significance of epidermal growth factor receptor (EGF-R) in human breast cancer: a review on 5232 patients. *Endocr Rev* **13**: 3–17

Kuenen-Boumeester V, Henzen-Logmans SC, Timmermans MM, van Staveren IL, van Geel A, Peeterse HJ, Bonnema J and Berns EM (1999) Altered expression of p53 and its regulated proteins in phyllodes tumours of the breast. *J Pathol* **189**: 169–175

Lakhani SR, Jacquemier J, Sloane JP, Gusterson BA, Anderson TJ, van de Vijver MJ, Farid LM, Venter D, Antoniou A, Storfer-lsser A, Smyth E, Steel CM, Haites N, Scott RJ, Goldgar D, Neuhausen S, Daly PA, Ormiston W, McManus R, Scherneck S, Ponder BA, Ford D, Peto J, Stoppa-Lyonnet D and Easton DF (1998) Multifactorial analysis of differences between sporadic breast cancers and cancers involving BRCA1 and BRCA2 mutations. *J Natl Cancer Inst* **90**: 1138–1145

Martin AM and Weber BL (2000) Genetic and Hormonal Risk Factors in Breast Cancer. *J Natl Cancer Inst* **92**: 1126–1135

Otterbach F, Bankfalvi A, Bergner S, Decker T, Krech R and Boecker W (2000) Cytokeratin 5/6 immunohistochemistry assists the differential diagnosis of atypical proliferations of the breast. *Histopathology* **37**: 232–240

Papelard H, de Bock GH, van Eijk R, Vliet Vlieland TP, Cornelisse CJ, Devilee P and Tollenaar RA (2000) Prevalence of BRCA1 in a hospital-based population of Dutch breast cancer patients. *Br J Cancer* **83**: 719–724

Paterson JW (1998) BRCA1: a review of structure and putative functions. *Dis Markers* **13**: 261–274

Peelen T, van Vliet M, Petrij-Bosch A, Mieremet R, Szabo C, van den Ouweland AM, Hogervorst F, Brohet R, Ligtenberg MJ, Teugels E, van der Luijt R, van der Hout AH, Gille JJ, Pals G, Jedema I, Olmer R, van Leeuwen I, Newman B, Plandsoen M, van der Est M, Brink G, Hageman S, Arts PJ, Bakker MM and Devilee P (1997) A high proportion of novel mutations in BRCA 1 with strong founder effects among Dutch and Belgian hereditary breast and ovarian cancer families. *Am J Hum Genet* **60**: 1041–1049

Perou CM, Jeffrey SS, van de Rijn M, Rees CA, Eisen MB, Ross DT, Pergamenschikov A, Williams CF, Zhu SX, Lee JC, Lashkari D, Shalon D, Brown PO and Botstein D (1999) Distinctive gene expression patterns in human mammary epithelial cells and breast cancers. *Proc Natl Acad Sci USA* **96**: 9212–9217

Perou CM, Sorlie T, Eisen MB, van de Rijn M, Jeffrey SS, Rees CA, Pollack JR, Ross DT, Johnsen H, Akslen LA, Fluge O, Pergamenschikov A, Williams C, Zhu SX, Lonning PE, Borresen-Dale AL, Brown PO and Botstein D (2000) Molecular portraits of human breast tumours. *Nature* **406**: 747–752

Petrij-Bosch A, Peelen T, van Vliet M, van Eijk R, Olmer R, Drusedau M, Hogervorst FB, Hageman S, Arts PJ, Ligtenberg MJ, Meijers-Heijboer H, Klijn JG, Vasen HF, Cornelisse CJ, van't Veer LJ, Bakker E, van Ommen GJ and Devilee P (1997) BRCA1 genomic deletions are major founder mutations in Dutch breast cancer patients. *Nat Genet* **17**: 341–345

Phillips KA, Andrulis IL and Goodwin PJ (1999) Breast carcinomas arising in carriers of mutations in BRCA1 or BRCA2: are they prognostically different? [see comments]. *J Clin Oncol* **17**: 3653–3663

Ross DT, Scherf U, Eisen MB, Perou CM, Rees C, Spellman P, Iyer V, Jeffrey SS, Van de Rijn M, Waltham M, Pergamenschikov A, Lee JC, Lashkari D, Shalon D, Myers TG, Weinstein JN, Botstein D and Brown PO (2000) Systematic variation in gene expression patterns in human cancer cell lines. *Nat Genet* **24**: 227–235

Sgroi DC, Teng S, Robinson G, LeVangie R, Hudson JR, Jr and Elkahloun AG (1999) In vivo gene expression profile analysis of human breast cancer progression. *Cancer Res* **59**: 5656–5661

Tomlinson GE, Chen TT, Stastny VA, Virmani AK, Spillman MA, Tonk V, Blum JL, Schneider NR, Wistuba II, Shay JW, Minna JD and Gazdar AF (1998) Characterization of a breast cancer cell line derived from a germ-line BRCA1 mutation carrier. *Cancer Res* **58**: 3237–3242

Welcsh PL, Owens KN and King MC (2000) Insights into the functions of BRCA1 and BRCA2. *Trends Genet* **16**: 69–74

Wilson CA, Ramos L, Villasenor MR, Anders KH, Press MF, Clarke K, Karlan B, Chen JJ, Scully R, Livingston D, Zuch RH, Kanter MH, Cohen S, Calzone FJ and Slamon DJ (1999) Localization of human BRCA1 and its loss in high-grade, non-inherited breast carcinomas. *Nat Genet* **21**: 236–240

*© 2001 Cancer Research Campaign*
